# Click-chemistry approaches to π-conjugated polymers for organic electronics applications

**DOI:** 10.1039/c6sc01832g

**Published:** 2016-06-27

**Authors:** Assunta Marrocchi, Antonio Facchetti, Daniela Lanari, Stefano Santoro, Luigi Vaccaro

**Affiliations:** a Laboratory of Green Synthetic Organic Chemistry , CEMIN – Dipartimento di Chimica , Biologia e Biotecnologie , Università di Perugia , Via Elce di Sotto, 8 , 06123 Perugia , Italy . Email: assunta.marrocchi@unipg.it ; Email: luigi.vaccaro@unipg.it; b Polyera Corporation , 8045 Lamon Avenue , Skokie , IL 60077 , USA; c Center of Excellence for Advanced Materials Research (CEAMR) , King Abdulaziz University , Jeddah , Saudi Arabia; d Northwestern University , 2145 Sheridan Road , Evanston , IL 60208 , USA; e Dipartimento di Scienze Farmaceutiche , Università di Perugia , Via del Liceo, 1 , 06123 Perugia , Italy

## Abstract

Given the wide utility of click-chemistry reactions for the preparation of simple moieties within large architecturally complex materials, this minireview article aims at surveying papers exploring their scope in the area of π-conjugated polymers for application in organic electronics to enable advanced functional properties.

## Abstract 2

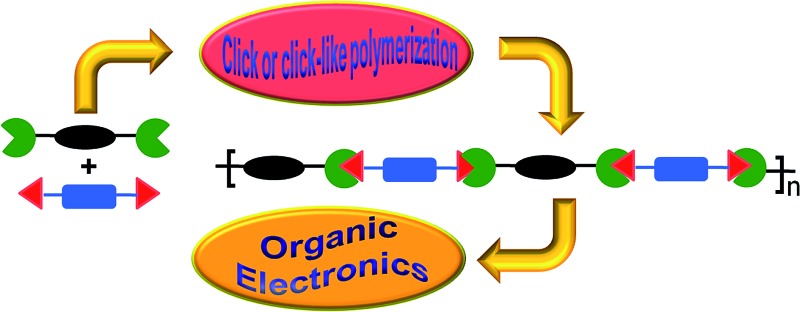
A survey of papers exploring the scope of click chemistry in the area of π-conjugated polymers for organic electronics is given.

Since the realization that π-conjugated polymers can be successfully implemented in several electronic and photonic devices the field of organic opto-electronics has grown exponentially.^1^ The most investigated devices are light emitting diodes (OLEDs), field-effect transistors (OFETs), sensors, integrated circuits, solar energy storage, photovoltaic cells (OPVs), laser diodes, and RF-ID tags.^1^ Organic non-volatile memory is another key area of application that exploits the advantages of organic materials.^2^ Typical methodologies to achieve photo/electro active polymers in organic electronics are based on traditional transition metal (TM) catalyzed cross-coupling reactions such as Stille, Suzuki, and Heck-type polymerizations (Scheme 1A and 1B).^3^

**Scheme 1:**
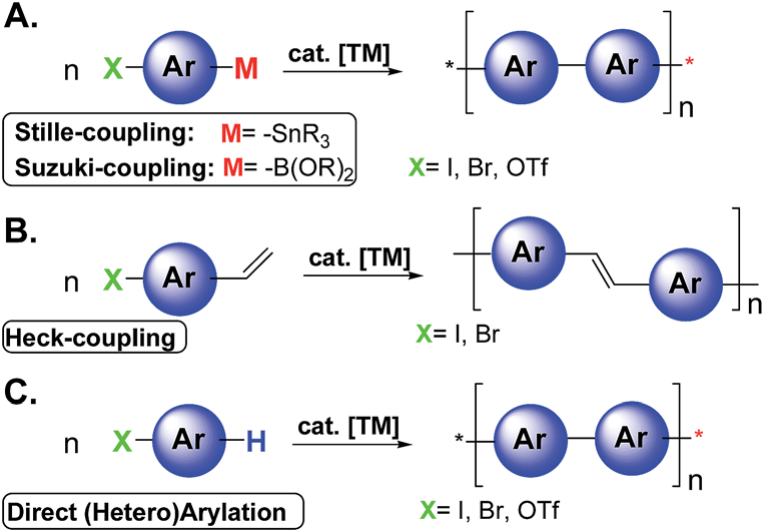
A general representation of polymerization using Stille, Suzuki, Heck and direct arylation cross-coupling reactions.

Despite their great versatility and wide substrate scope, these reactions may present drawbacks including the number of steps to synthesize the proper monomers, instability of organometallic reagents, poor conversion related to unreactive monomers, difficulties in controlling the polymer architecture, and poor atom economy, due to the formation of stoichiometric amounts of (toxic) byproducts. Moreover, electronic grade organic materials generally require extensive purification processes to remove undesired traces of byproducts and/or metals.^4^

Recently, the direct arylation methodologies (Scheme 1C) have received increasing attention appearing to be a simple and environmentally benign alternative to traditional cross-coupling reactions.^3,5^

In this context, efforts for implementing efficacious and green novel approaches to organic semiconductors which minimize the use of solvents and reagents, as well as the number of workup procedures,^6^ undoubtedly represent important contributions for the development of the field.

Click chemistry^7^ shares with green chemistry some of the most fundamental principles, by means of which more efficient and environmentally benign synthetic protocols can be designed and implemented. The concept of “click chemistry” was first introduced by Sharpless^8^ in 2001. A set of stringent criteria must be met by a chemical process to be classified as “click-type”. Among the most important, the process should be (i) able to generate inoffensive by-products removable by non-chromatographic methods, (ii) carried out under simple reaction conditions, (iii) “spring-loaded” for a single trajectory, *i.e.* characterized by a high thermodynamic driving force that drives it quickly and irreversibly to high yield of a single reaction product, with high reaction specificity (iv) based on readily available starting materials and (v) without the use of an additional reaction medium or only using benign solvents.^8^ The copper(i)-catalyzed cycloaddition of azides and alkynes to give 1,2,3-triazoles (CuAAC) is the flagship of click chemistry. The uncatalyzed reaction, *i.e.* the Huisgen 1,3-dipolar cycloaddition of azides and alkynes,^9^ proceeds very slowly even at high temperatures and gives a mixture of 1,4- and 1,5-substituted 1,2,3-triazoles (Scheme 2A).

**Scheme 2:**
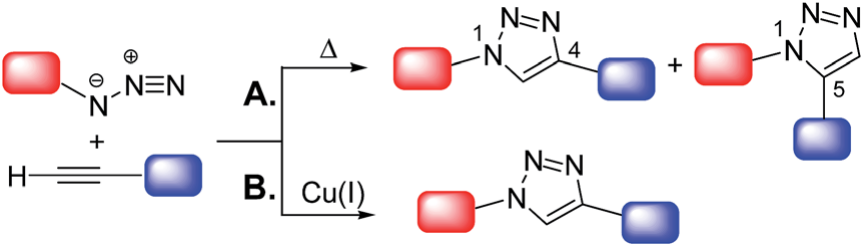
Synthesis of 1,2,3-triazoles *via* 1,3-dipolar cycloaddition of azides and terminal alkynes.

However, in the presence of a source of catalytically active copper(i) species^7*b*^ the reaction is markedly accelerated as shown independently for the first time in 2002 by the research groups of Sharpless^10^ and Meldal.^11^ Moreover, the Cu(i)-catalyzed process occurs regioselectively, affording exclusively the 1,4-disubstituted isomer (Scheme 2B). It should be also noted that in alternative to Cu(i)-catalyzed process, some classes of 1,2,3-triazoles can be also prepared *via* very efficient organocatalytic methods.^12^

Several studies have been reported^13^ to elucidate the mechanistic pathway for CuAAC, on the basis of experimental evidence and density functional theory (DFT) calculations. The most recent experimental mechanistic study by Fokin and coworkers^13*a*^ outlined in Scheme 3 demonstrated unambiguously the participation of a dinuclear copper intermediate.

**Scheme 3:**
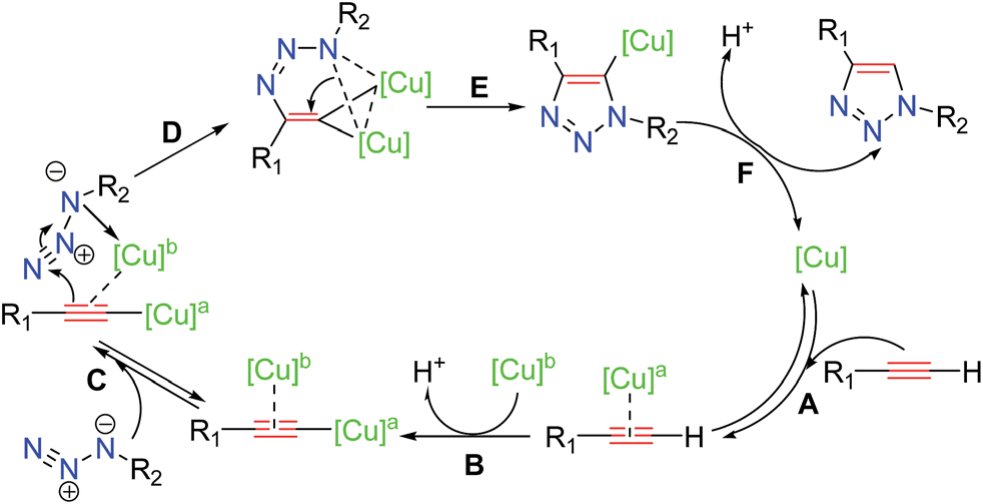
Most recent CuAAC mechanism proposed by Fokin and co-workers.

Since then, click chemistry have found widespread application in various research areas, including the syntheses of dendrimers^14^ and rotaxanes,^15^ drug discovery,^7,16^ biochemistry,^7,16b–d^ and for the chemical modification of surfaces and nanostructures.^7^

The utility of click reactions in polymer chemistry has also been explored. Although most studies reported post-functionalization of pre-formed polymers,^7,17^ recent efforts employed these reactions as efficient polymerization techniques (click polymerization).^7d,e,g,18^ The azide–alkyne click reaction, in particular, holds the promise to become a powerful synthetic tool to develop unprecedented functional materials which are expected to open new opportunities for organic electronics/photonics applications.

For instance, the triazole ring may serve as electron-accepting unit to impart optical nonlinearity to a given system.^19^

Triazole derivatives may also display the unique characteristic of aggregation-induced emission (AIE),^18f,g,20^ that is, their emission intensity may be enhanced in the solid/aggregate states, making them particularly desirable for applications as advanced photonic materials.

Although several studies indicate that the conjugation in triazole derived structures is limited through the triazole moiety,^21^ in the last decade a number of interesting examples demonstrated extended π-electron systems incorporating the 1,2,3-triazole moiety into the conjugation path.

Triazole-containing molecular systems may function as electron or even ambipolar^22^ transporting materials in the fabrication of organic light-emitting diodes. Moreover, Ratner, Mirkin and coworkers^23^ recently demonstrated that the triazole ring maintained the conjugation required for electronic transport in molecular transport junctions to bridge nanogaps, thereby enabling the creation of nanoelectronic devices with diverse functions and applications. Finally, Guldi and co-workers^24^ clearly demonstrated that aromatic 1,2,3-triazoles may represent excellent conjugating π-linkers for rapid and efficient photoinduced electron transfer between remote electron donor and electron acceptor moieties, namely zinc porphyrin and C_60_, respectively. This is an important factor for applications of organic materials in solar energy storage and photovoltaic devices.^25^ Similar conclusions have been drawn independently by several other groups,^19b,20,26^ when exploring the use of 1,2,3-triazoles to bridge donor–acceptor groups featuring different substitution patterns.

In this review article, we have surveyed seminal papers exploring the utility of the click reaction in the area of π-conjugated polymers for organic electronics. We have mainly focused the discussion on conjugated hetero-structures from azide–alkyne precursors. We have also briefly surveyed reactions other than CuAAC featuring the essential “click” attributes and that have been applied for the preparation of π-conjugated macromolecules. The key physical and morphological properties of the resulting materials as well as their performances in organic electronic devices have been discussed.

Several interesting studies^27^ have been carried out on the use of azide–alkyne click reaction as an effective strategy for tuning the associated self-assembly properties in the post-functionalization of pre-formed conjugated polymers. However, a survey of these methods is beyond the scope of this review.

The first report on the synthesis of conjugated polymers by the Cu(i)-catalysed 1,3-dipolar “click” reaction was published in 2005 by van Maarseveen, Reek and coworkers^28^ who synthesized linear poly(triazole)s **P1–P3** *via* polymerization of 2,7-diazidofluorene **1** and aromatic diynes **2–4** (Scheme 4).

**Scheme 4:**
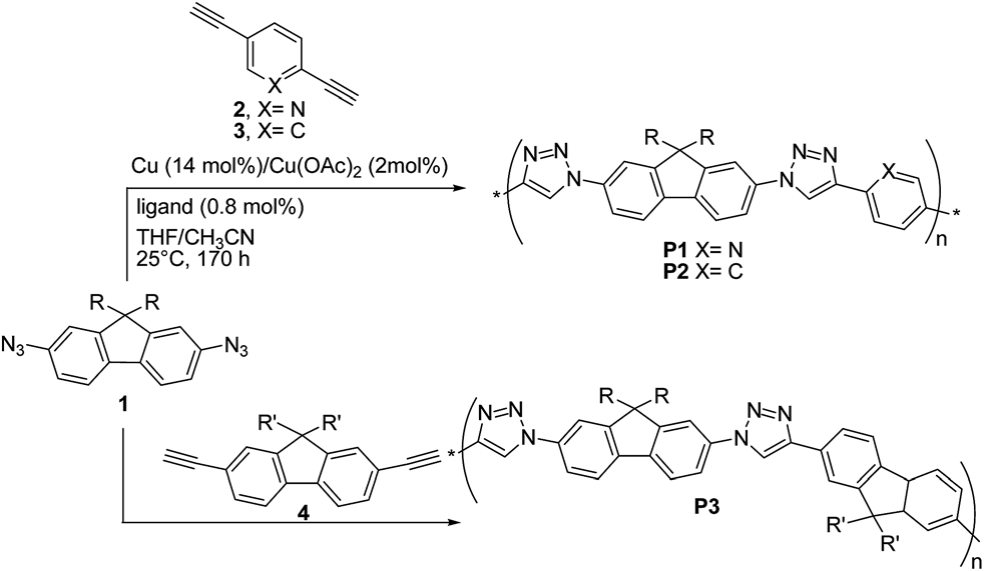
Synthesis of “click” polymers **P1–P3**.

The reaction between **1** and 2,5-diethynylpyridine **2** was performed in THF/CH_3_CN, by employing a 1 : 1 co-monomer molar ratio, Cu (∼14 mol%)/Cu(OAc)_2_ (∼2 mol%) as the catalyst and *tris*-(benzyltriazolylmethyl)amine (∼0.8 mol%) as the ligand. Copolymer **P1** was obtained with a *M*_n_ up to ∼25 kDa (PDI = 1.9). Under the same conditions, copolymers **P2** and **P3** were obtained with *M*_n_ up to 327 kDa (PDI = 1.21) and *M*_n_ = 8 kDa (PDI = 1.61), respectively.

Interestingly, when the reaction between **1** and **2** was carried out at –10 °C for 65 h, **P1** molecular weight was higher (*M*_n_ = 20.6 kDa, PDI = 2.86) than that of the polymer obtained at 25 °C for 170 h, thus suggesting an exothermic polymerization reaction. Unfortunately, no reaction yields were reported. The fluorene-containing copolymer absorption spectra were found to be the superposition of those of the monomers. Furthermore, the authors observed that **P1–P3** were highly emissive in THF, with photoluminescence peaks located at 360–380, *i.e.* the emission was dominated by the fluorene units. The highest quantum yield (*Φ*_F_ = 55%) was achieved for **P3**. These findings indicate a poor electronic communication between the polymers aromatic building blocks. However, cyclic voltammetry measurements on a model compound consisting of two pyridyl-triazole units bridged by a fluorene unit (*i.e.* the product between **1** and **2**) showed one two-electron reduction (–1.86 V) but no oxidation, suggesting that the present materials might be useful semiconductors.

Shortly thereafter, Bunz and coworkers^29^ prepared the conjugated poly(triazole)s **P4–P9** (Scheme 5) by reacting 2,7-diazidofluorenes **1** (or **5**), and 4,4′-diazido-3,3-dimethoxy-biphenyl **6** with 2,5-dialkyl-1,4-diethynyl benzenes **7** and **8**, in the presence of CuSO_4_ (5 mol%)/sodium ascorbate (SA).

**Scheme 5:**
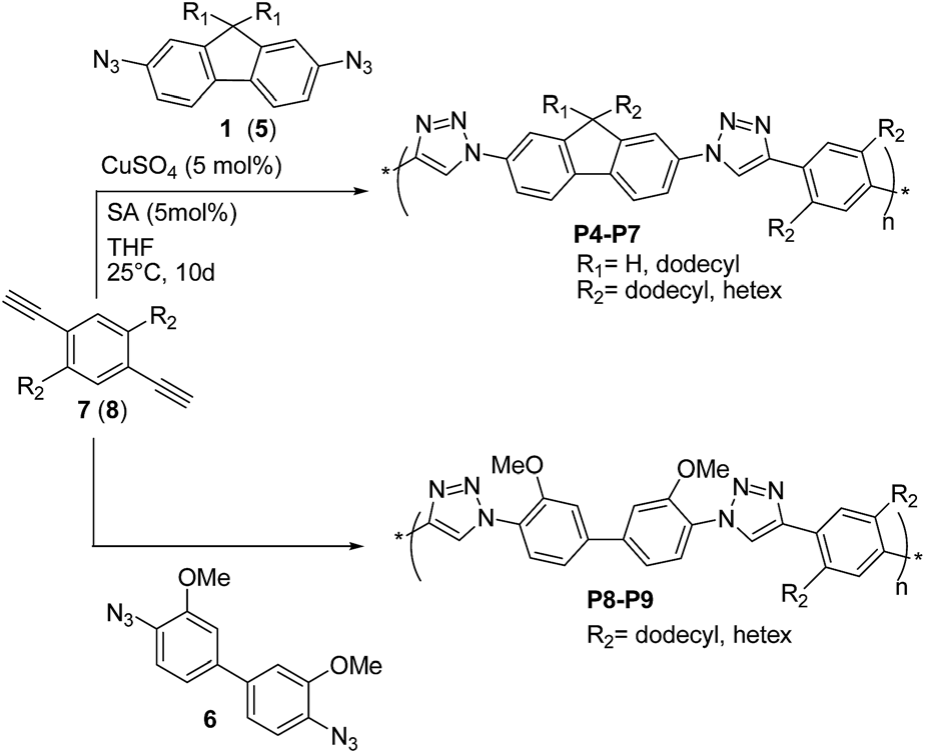
Synthesis of “click” polymers **P4–P9**.

These copolymers were obtained in high yields (80–92%), with a maximum *M*_n_ value of 8.7 kDa achieved for **P7** (PDI = 5.8). According to ^1^H- and ^13^C-NMR spectroscopy, all poly(triazole)s **P4–P9** exhibited regioregular 1,4-substitution of the triazole unit and showed blue fluorescence in solution, with the highest quantum yields (*Φ*_F_ ∼ 40%) achieved for **P7** and **P9**. On the other hand, no fluorescence was observed in the solid state, which was attributed to aggregation phenomena.^30^ The optical properties were independent from **P4–P9** molecular weights, thereby suggesting that the used building blocks led to species with localized HOMO/LUMO orbitals. This was also corroborated by quantum mechanical calculations on the model compound **P8**. Interestingly, fluorescence measurements and theoretical calculations suggested that^29^ protonation at the 3-position of the triazole group in **P8** resulted in the decrease of HOMO–LUMO gap and more delocalized frontier molecular orbitals. On these basis, the use of alkyne and azide precursors substituted with electron-donating and electron-withdrawing groups, respectively, is expected to further lower the band-gap of **P4–P9**, which may become of interest as semiconductors.

Finally, by using a heated tip of an AFM cantilever (∼225 °C) the authors^29^ succeeded in writing crisp nanoscale features into **P7** thin-films. The absence of tackiness and ripping led the authors to the conclusion that these materials were attractive as novel semiconductors that could have been easily thermally structured.

Lee, Jin and coworkers^31^ reported the preparation of π-conjugated soluble poly(triazole)s **P10–P12** (Scheme 6) by click polymerization (∼90% yields) of 2,7-diazido-9,9-dioctylfluorene (**9**) with 2,7-diethynyl-9,9-dioctylfluorene (**10**), 4,7-diethynylbenzothiadiazole (**11**), and 2,7-diethynylcarbazole (**12**), respectively, employing a 1 : 1 co-monomer molar ratio and CuSO_4_·5H_2_O/sodium ascorbate/triethylamine as the catalytic system.

**Scheme 6:**
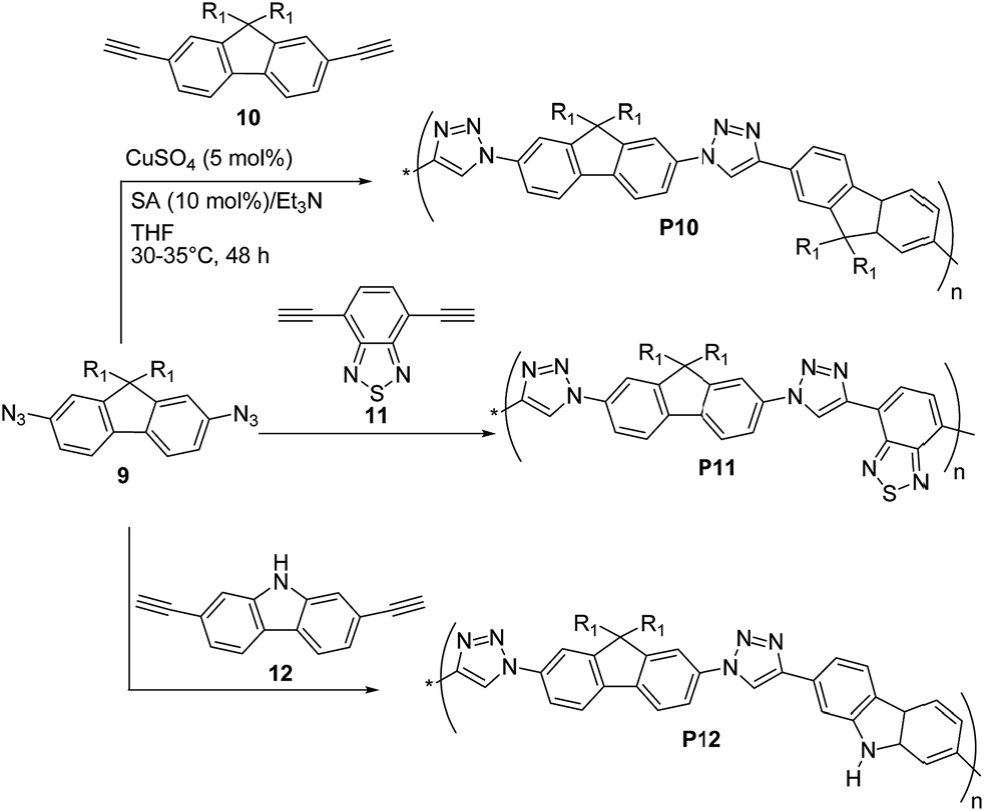
Synthetic routes for the “click” polymers **P10–P12**.

An average *M*_n_ as high as ∼8.3 kDa was obtained for **P10** (PDI = 1.92) whereas **P11** and **P12** both exhibited lower *M*_n_ ∼ 6 kDa (PDI = 1.38 and 1.92, respectively). Polymers **P10–P12** were found to be stable up to 300 °C (*via* TGA) and exhibited optical absorption maxima in CHCl_3_ in the range of 328–350 nm, with **P10** being the most red-shifted. Similar UV-vis absorption spectra were recorded in the solid state. The well-structured PL spectra of **P10–P12** in solution revealed a blue light emission between 370 and 406 nm, whereas their emission spectra in the solid state were slightly red-shifted (15–40 nm). The HOMO and LUMO energy levels of **P10** are –5.23 eV and –3.25 eV, respectively, whereas a HOMO of –5.39 (5.35) eV and a LUMO of –2.44 (3.14) eV were found for **P12**, on the basis of cyclic voltammetry and optical absorption data.

More recently, Nesterov and coworkers^32^ reported an interesting stepwise methodology based on surface-initiated Cu(i)-catalyzed click polymerization to synthesize “brush” polymer **P13** (Scheme 7). It is noteworthy to highlight that this approach allows the access to surface-grafted (“brush”) polymers which are an interesting alternative for high-performance, long-term operation organic electronics, possibly helping charge injection and charge transport processes which are crucial for many devices.^33^

**Scheme 7:**
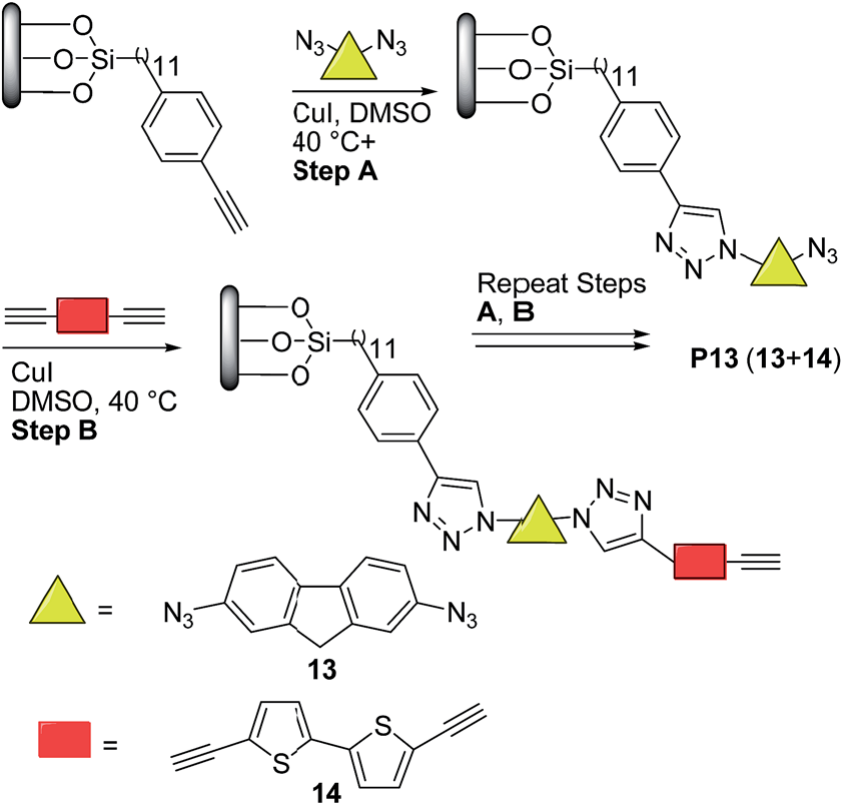
Preparation of semiconducting polymer thin films *via* surface-initiated stepwise “click” polymerization.

Thus, the authors^32^ functionalized a quartz substrate with a monolayer of trimethylsilyl-acetylene initiator. Next, the activated surface was immersed in a dimethyl sulfoxide (DMSO) solution of the bis-azide monomer **13** (Scheme 7) in the presence of 10 mol% CuI, followed by rinsing. Finally, the substrate was placed into a DMSO solution of the bisacetylene monomer **14** and 10 mol% CuI, followed by rinsing. This sequence was repeated for 34 times. Note here that the initially prepared monomer solutions could last for the entire duration of polymerization, thereby avoiding wasting monomers. Moreover, no oligomer/polymer formation was detected in these solutions, which indicated that a controlled stepwise polymerization occurred only on the substrate surface.

Atomic force microscopy (AFM) data revealed that **P13** films exhibited a thickness of ∼28 nm and a surface morphology (rms ∼ 6 nm) featuring uniform cylindrical domains of ∼90 nm in diameter with tight packing density (Fig. 1). The domain morphologies were investigated by polarization-dependent ultraviolet photoemission spectroscopy (UPS) and found to be oriented normally to the substrate protruding throughout the film. Furthermore, the authors claimed that each domain likely featured a uniform, well-ordered packing of the macromolecules. UV-vis thin-film absorption spectrum exhibited a maximum at ∼350 nm, with a broad band spanning to almost 700 nm, as well as an optical band gap of 2.52 eV. The HOMO and LUMO energy levels for **P13** were found to be –5.28 eV and –2.76 eV, respectively, on the basis of cyclic voltammetry experiments and optical absorption data.

**Fig. 1:**
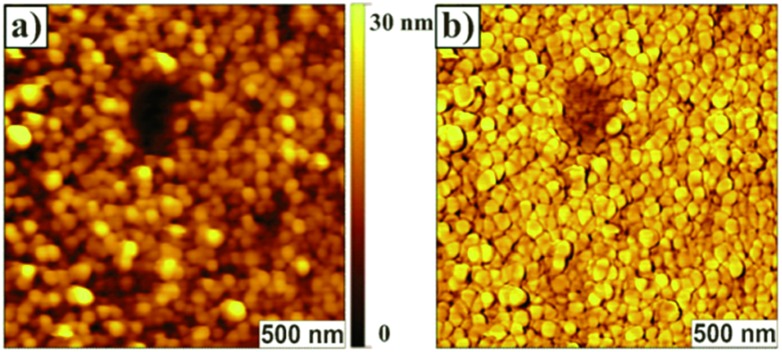
Surface morphology imaged by contact mode AFM. (a) Topograph of the film of **P13** prepared on a quartz surface (roughness, rms, ∼6 nm); (b) corresponding lateral force image. Centre area has a trench in the film made by ‘‘nanoshaving’’ to show continuity of the cylindrical domains. Reproduced with permission.^32^ Copyright 2011, The Royal Society of Chemistry.

The potential of ‘click’ polymers from azide–alkyne precursors as conducting molecular wires has been demonstrated by Luo *et al.* ^34^ More specifically, the authors prepared oligophenylenetriazole wires **O1** and **O2** (Scheme 8) having systematically varied lengths up to 10 nm. The **O1** and **O2** wires were built from Au surface by the general route exemplified for the synthesis of **O2** in Scheme 8.

**Scheme 8:**
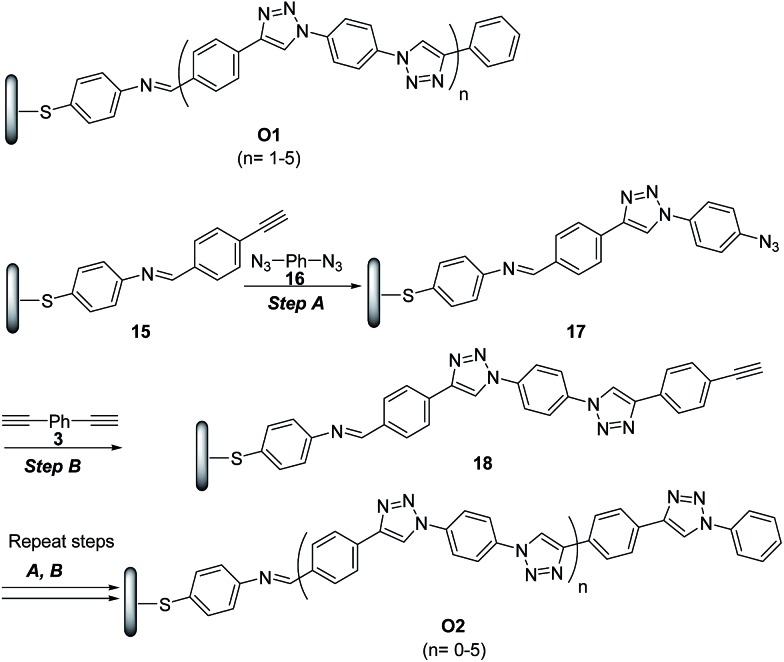
Synthetic route to oligophenylenetriazoles **O1** and **O2**. (A) CuSO_4_·5H_2_O (2 mmol%), SA (15 mmol%), EtOH/H_2_O (2.5 : 1), rt; (B) CuSO_4_·5H_2_O (2 mmol%), SA (15 mmol%), CH_3_CN/H_2_O (2.5 : 1), rt.

Electrical measurements of oligophenylenetriazole wires were performed by conducting probe atomic force microscopy (CP-AFM) and, interestingly, their current–voltage characteristics were reported to be similar to other conjugated wire molecules,^35^ with a transition from tunnelling to hopping transport as wire length increased.

Bäuerle and co-workers^36^ successfully employed the click chemistry approach to synthesize a range of thiophene-based oligomers of a donor–acceptor type **O3**, in which the thiophene moiety is the donor (electron-rich) moiety and 1,2,3-triazoles are the acceptor (electron-poor) units. These systems were designed bearing in mind that a 1,2,3-triazole can act as a weak electron-acceptor.^19*b*^ Excellent yields up to 99% were obtained by reacting an equimolar mixture of the corresponding terminal acetylenes and *in situ* generated organic azide. Optimal conditions involved the use of copper(i) iodide (10 mol%)/sodium ascorbate (10 mol%)/*N*,*N*′-dimethylethylenediamine (DMEDA, 20 mol%) as the catalytic system in ethanol–water (50 °C, 20 h) (Scheme 9). Notably, a conjugation through the triazole ring was generally operative, as indicated by the spectroscopic and redox properties of the above systems. These data points out again the importance of designing suitable substrates in such approach to limit the charge trapping characteristics of the triazole ring.

**Scheme 9:**
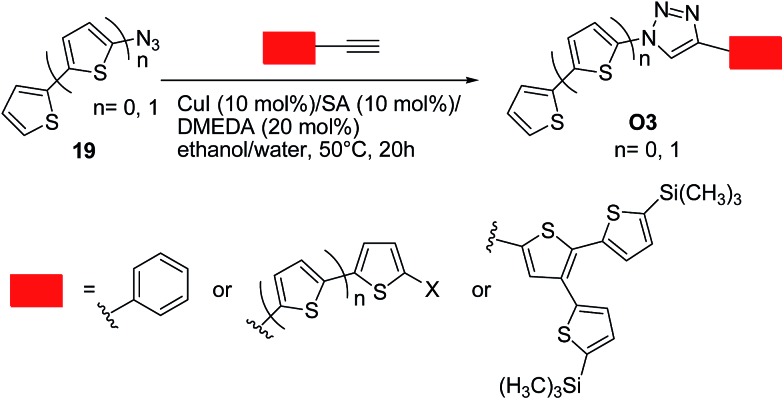
Synthesis of 1,4-disubstituted 1,2,3-triazoles **O3** from azides **19** and corresponding terminal acetylenes.

Very recently Song *et al.* ^37^ demonstrated that triazole rings are advantageous units to high performance electrical memory polymers, in view of the high hole affinity of the three nitrogens with lone-pair electrons. A series of brush polystyrenyl (PS) derivatives **P14–P16** (Scheme 10) bearing the triazole moiety in their bristle were prepared in high yield (up to 98%) employing as a key step the click reaction of a poly(4-azidomethylstyrene) **20** with 4-[(4-ethynylphenyl)ethynyl]-*N*,*N*-dihexadecylaniline **21**. Next, the authors investigated the triazole containing polymers electrical memory characteristics by incorporating them as active layer into devices with aluminium top and bottom electrodes (Fig. 2).

**Scheme 10:**
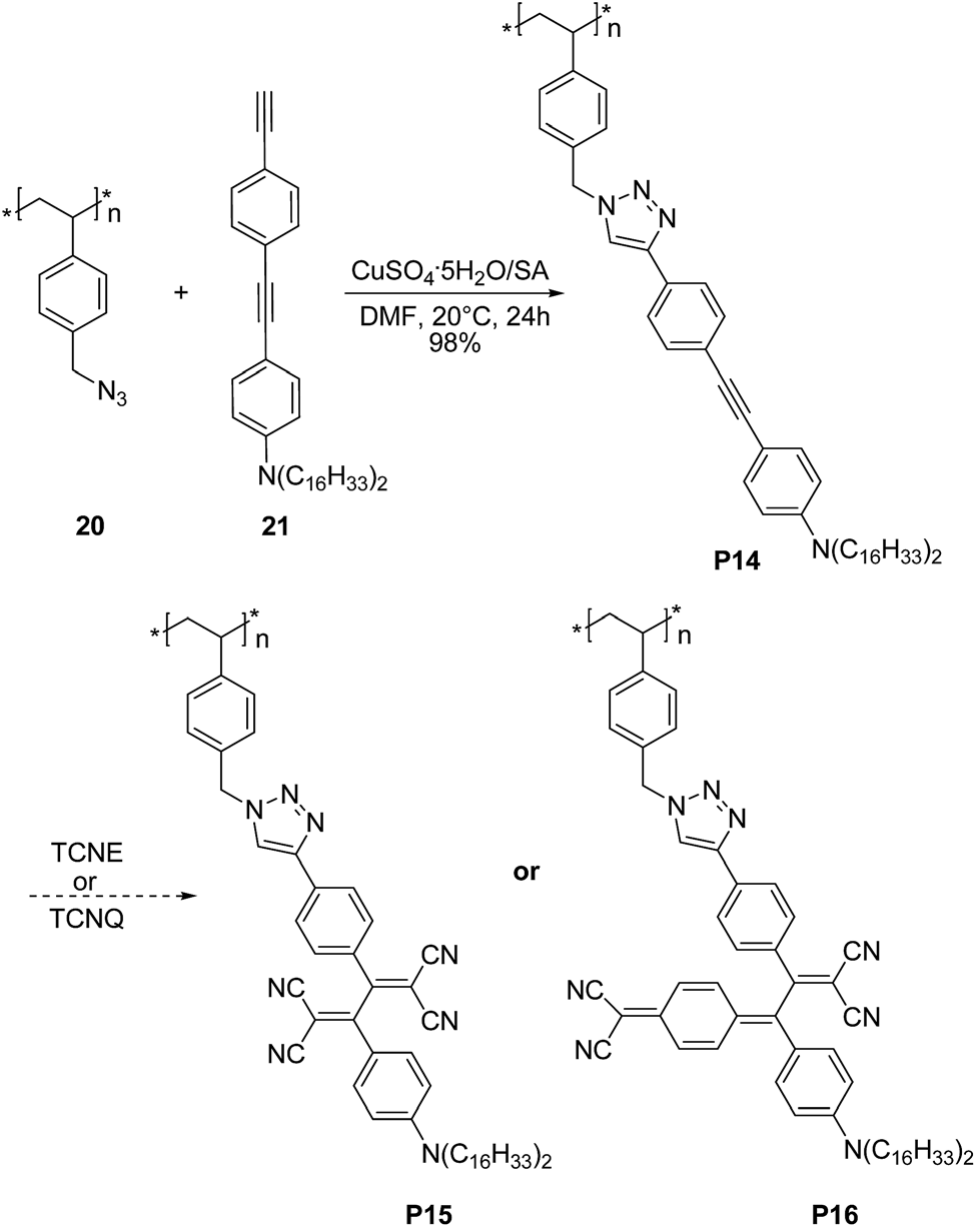
Synthetic route to **P14–P16**. TCNE and TCNQ stand for tetracyanoethylene and 7,7,8,8-tetracyanoquinodimethane, respectively.

**Fig. 2:**
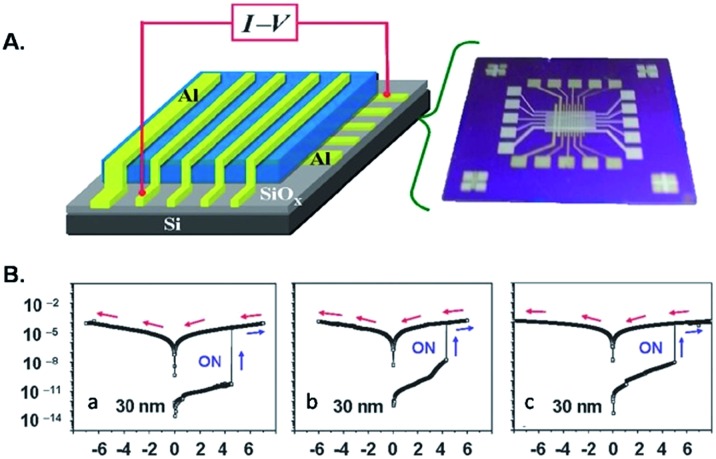
(A) Device structure and (B) representative *I*–*V* curves of the devices incorporating (a) **P14**, (b) **P15**, and (c) **P16** 30 nm thick films [adapted from ref. 37].

Interestingly, **P14–P16** revealed to be highly suitable for the production of unipolar permanent memory devices that can be operated with very low power consumption, a high ON/OFF current ratio and high stability and reliability. Furthermore, the memory type could be tuned from p-type (**P14**) to n-type (**P15**, **P16**) by the incorporation of a strong electron accepting TCNE or TCNQ moiety into the ethynylphenyl unit linked to the triazole moiety, as a result of a reduced LUMO level with regard to the electrode work function.

Apart from the combination of the azide–alkyne functionalities, other reactions based on “spring-loaded” reactants and featuring the essential “click-chemistry” attributes have been exploited to synthesize π-conjugated macromolecules for organic electronics applications.

For instance, Jørgensen and Krebs^38^ and, more recently, Eichen and coworkers^39^ developed a stepwise directional synthetic route (Fig. 3) to oligo-arylene-vinylenes.

**Fig. 3:**
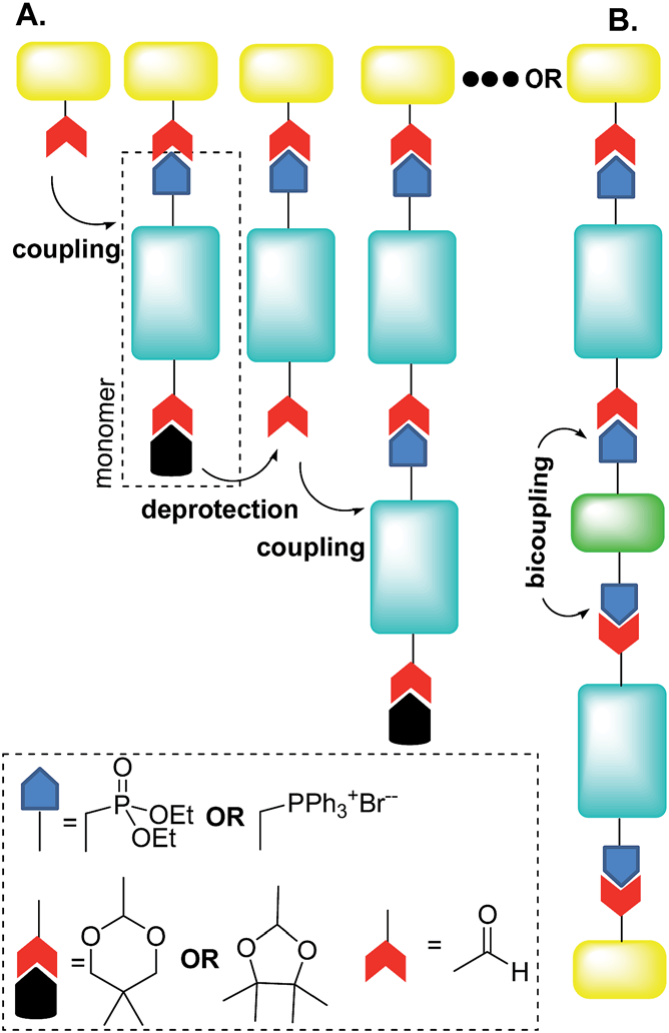
Schematic illustration of Jørgensen^38^ and Eichen^39^ “click” approach to oligo-arylene-vinylenes. Adapted with permission.^39*b*^ Copyright 2011, Wiley-VCH.

Oligomers of arylene-vinylenes are of interest in many areas of materials research, in some cases as models for the corresponding poly-*p*-phenylene-vinylenes (PPVs) that are used in organic light emitting diodes, field effect transistors and photovoltaics.^1^

Most remarkable the development of such alternative reaction scheme by the Jørgensen group^38^ was prompted by the observation that when palladium catalyzed reactions were used for polymerization, incorporation of small palladium nanoparticles was inevitable. As a consequence these metal residues were very difficult or impossible to remove, substantially affecting the device performance.^4^

Thus, the authors employed only one monomer featuring two different terminal functionalities. On a stilbene core, it was introduced a phosphonate ester group at one extremity and an acetal-protected aldehyde at the other (Fig. 3A). The oligomerization then started with the Horner–Wadsworth–Emmons (HWE) reaction between an aldehyde and the monomer in the presence of potassium *t*-butoxide.

The product of this HWE reaction was an end-capped “monomer” that was subsequently subjected to the deprotection of the aldehyde in the presence of dilute hydrochloric acid, as depicted in Fig. 3A. Oligomerization then proceeded by alternating reaction of the previous aldehyde-terminated *p*-phenylenevinylene fragment with the monomer and deprotection of the acetal.

A series of oligomers **O4** (Fig. 4) featuring up to 11 phenylene-vinylene units were successfully prepared in good to high yields (57–96%). Interestingly the authors demonstrated that the proposed synthetic scheme gave easy access to oligomers of high purity.

**Fig. 4:**
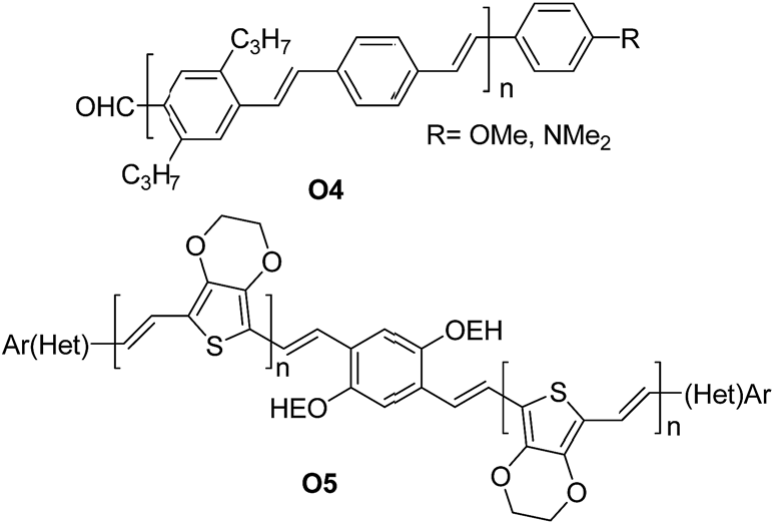
Examples of π-conjugated “click” oligo-arylene-vinylenes reported by Jørgensen *et al.* ^38^ and Eichen *et al.* ^39^

Subsequently, the oligomers could be further derivatized easily at the aldehyde position to create a series of systems with a range of electron-accepting or electron-donating substituents. Photovoltaic cells using the structure of ITO/PEDOT:PSS/**O4**/Al were assembled. Illuminated under simulated sunlight (AM1.5) gave short circuit currents (*I*_sc_) in the range of 0.015–0.5 mA cm^–2^ and typical open circuit voltage (*V*_oc_) of 0.4–0.8 V. The maximum efficiency obtained was ∼0.1%.

Eichen and coworkers^39^ applied “click”-type approaches depicted in Fig. 3 to produce a wide range of structurally controlled functionalized oligo arylenevinylene systems, including **O5**. The general process used bifunctional monomers consisting of a π-conjugated backbone bearing two functional groups, *i.e.* a phosphine/phosphonate group and an acetal-protected aldehyde. The first step in the arylene-vinylene formation was a Wittig–Horner reaction between a functionalized aldehyde (so-called start unit) with the *in situ* generated ylide of a bifunctional monomer (using *t*-BuOK). Next, the product was deprotected to release the aldehyde group of the di-arylenevinylenes for the subsequent step. This sequence has been repeated several times, using the different bifunctional monomers to construct the oligomer with the desired sequence. Additionally, at a certain stage, the deprotected oligomer has been coupled with a bifunctional system bearing two phosphine/phosphonate groups, producing symmetrical oligo-phenylene-vinylenes (Fig. 3B).

Optical absorption/fluorescence spectroscopy and cyclic voltammetry were extensively used to characterize the electronic structure of the arylenevinylene systems, thereby highlighting the tuneability of the optical and HOMO/LUMO band positions and, ultimately, showing the potential inherent to such approach.

Next, organic field-effect transistors and light emitting diodes were prepared from selected materials, and field-effect mobilities (*μ*) up to ∼1 × 10^–3^ cm^2^ V^–1^ s^–1^ as well as CIE chroma coordinates (*x*, *y*) = (0.6354, 0.3625) were achieved.

Moreover, very recently, Demissie *et al.* ^40^ and Smith *et al.* ^41^ reported the successful synthesis of π-conjugated molecular wires on Au surfaces using imine click-like (condensation) reaction to ensure high yields (∼99%) in a simple sequential monomer addition process. The reaction schematic in Scheme 11 demonstrates the alternate addition of benzene-1,4-dicarbaldehyde **22** (ref. 40) (or thiophene-2,5-carboxyaldehyde **23**)^41^ and 1,4-diaminobenzene **24** monomers to give **O6** and **O7**-type oligomers. The wires ranged in length from 0.6 to 5.5 nm. Electrical transport measurements were carried out for **O7**-type oligomers,^41^ revealing that charge transport in short oligomers was temperature-dependent whereas for longer ones it is activated, consistent with a crossover from tunneling to hopping transport. Interestingly, optical and electrochemical measurements indicated that for wires featuring more than three repeating units charge is not delocalized across the entire wire length.

**Scheme 11:**
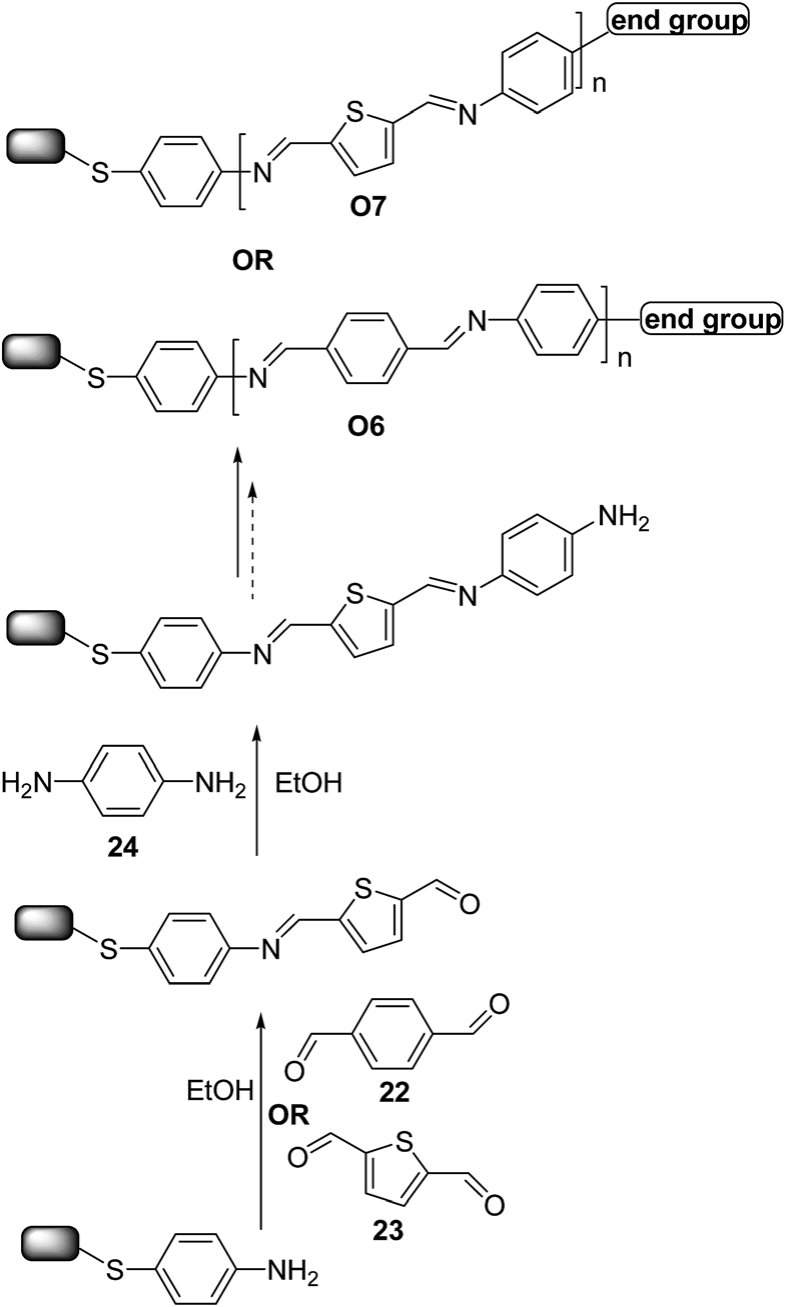
Reaction schematics for the molecular wires **O6** and **O7** self-assembled on Au surface.

Research efforts have been also devoted to exploit thiol-yne click^42^ reaction to synthesize electronically active π-conjugated polymers. Indeed, since the thiol-yne reaction allows simple addition of two thiol groups to an alkyne, it appears to be perfectly suitable to enable multifunctional conjugated structures. For instance, Tang and co-workers developed the first example of Rh-catalyzed thiol-yne click polymerization for the synthesis of a series of linear poly(vinylenesulphide)s (PVSs), including **P17a** and **P17b** (Scheme 12).^43^ The Rh(PPh)_3_Cl catalyzed click polymerization of dithiol **25** and diynes **26a**,**b** was carried out under mild conditions at room temperature, leading to the corresponding sulphur rich polymers **17a** and **17b** in high yields (∼85–92%) and good-to-high molecular weight (*M*_w_ = 13.3 kDa, PDI = 3.4 and *M*_w_ = 7 kDa, PDI = 3.2, respectively). Polymer **17a** featured mixed isomers with *E*/*Z* ratio of 50 : 50 whereas when the ferrocene-containing dyine **26b** was employed, a PVS with high *E* content (∼90%) was obtained. In a further contribution^44^ the same group reported a non-metallic catalyst-mediated click polymerization of dithiol **25** and a series of dipropiolates, including **26c** (Scheme 12). The reaction was carried out at room temperature (24 h), and readily provided the corresponding PVS **17c** with high molecular weight (*M*_w_ = 21 kDa, PDI = 2.9) and a predominant *Z* configuration (*Z*/*E* = 78%) in a satisfactory yield (73.5%). Tang and co-workers succeeded^45,46^ also in establishing a catalyst-free thiol-yne click polymerization and preparing conjugated linear and hyperbranched PVSs (*e.g.* **P17d–f**, Scheme 12, and **P18**, Scheme 13, respectively). The polymerizations of the aromatic dithiol **25** and diynes **26d–f** or **27** in equimolar ratio could be performed under very mild conditions (THF, 30 °C), without any additive. Importantly, this polymerization was quite efficient (78–97% yield) and PVSs with *M*_w_ up to 61 kDa (**P18**, PDI = 4.96) could be obtained. The *E*/*Z* ratio generally resulted to be ∼50/50. Interestingly, the AIE active^20a,d^ tetraphenylethylene moiety containing PVSs, *i.e.* **P17c**, **P17f** and **P18** were also found to possess the AIE feature.

**Scheme 12:**
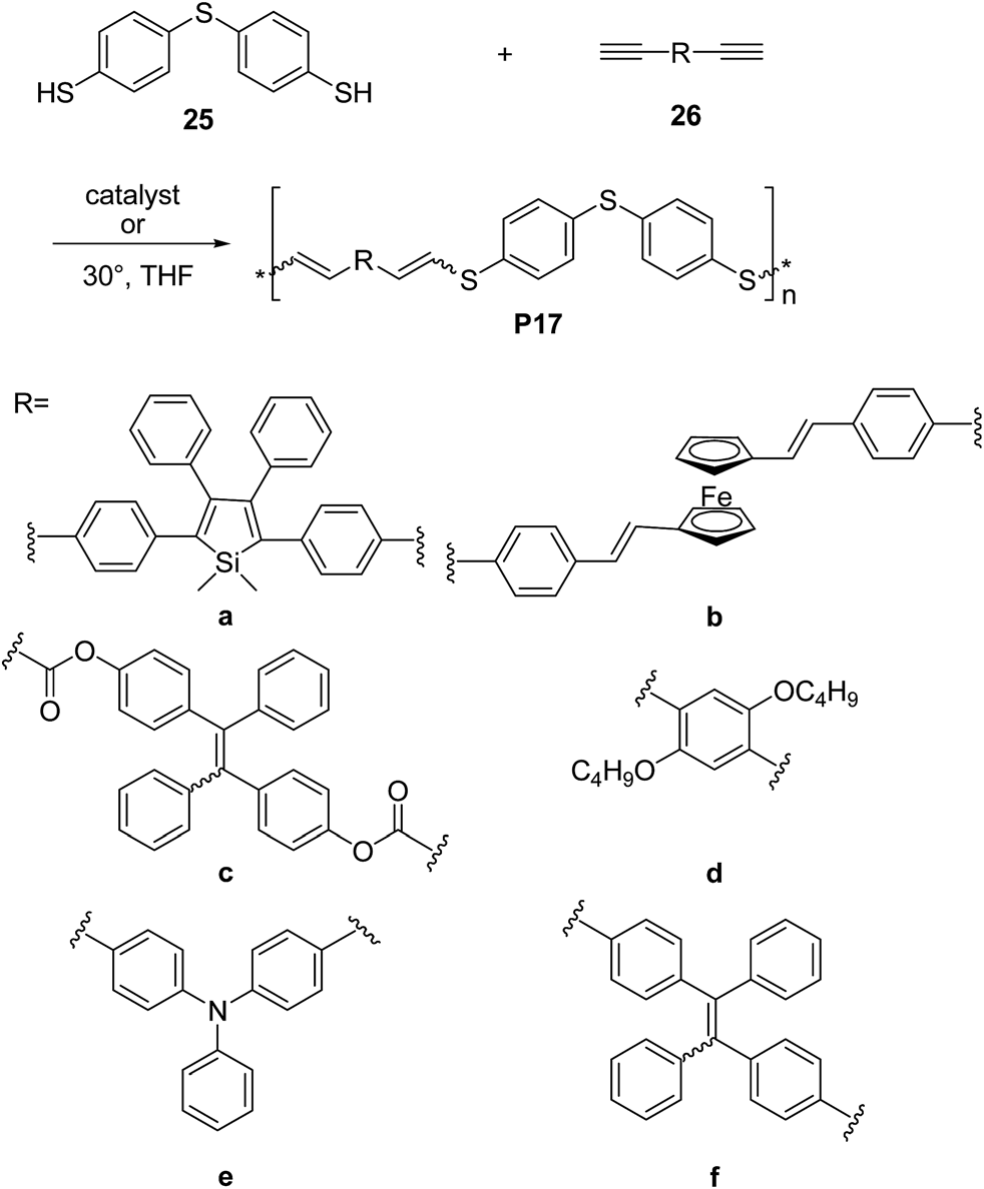
Syntheses of poly(vinylsulfide)s **P17** *via* thiol-yne click polymerization reactions of monomers **25** and **26**.

**Scheme 13:**
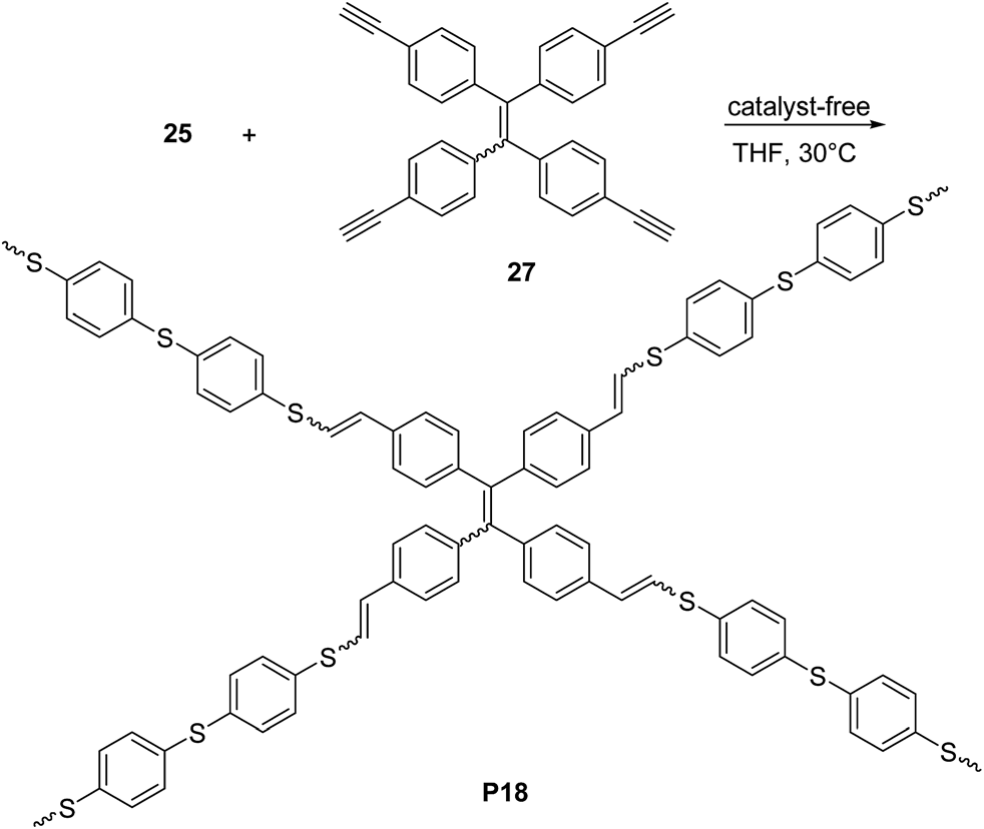
Synthesis of hyperbranched π-conjugated polymer **P18** by catalyst-free thiol-yne reaction between monomers **25** and **27**.

## Conclusions

In conclusion, click chemistry may provide an efficient and versatile way for the synthesis of structurally diverse π-conjugated polymers/oligomers. Particularly, the CuAAC reaction continues to confirm its role as a powerful synthetic tool contributing to unaccountable applications. It also proves to be very effective for the synthesis of opto-electronic materials opening new opportunities for organic electronics applications. As inferred from the number of contributions emerged in the last few years, the “click” polymerization is yet far to be fully exploited in this field. Nevertheless, the examples of conjugated triazole-based materials reported to date have highlighted the great potential for new directions, identifying a new family within organic materials. The ultimate scope of this review is to anticipate the need of future research in this area. A major issue that need to be addressed in the near future is to improve the experimental conditions to allow a (CuAAC) click polymerization reaction to proceed efficiently without catalyst or in such a way that copper can be removed completely. Indeed, although there are many reports^7d,h,20b,42c^ on metal- or catalyst-free azide–alkyne cycloadditions and other click reactions, only few papers^44–46^ have addressed this issue for the preparation of opto-electronic polymers. Additionally, there are examples^7a,b^ reporting the utilization of supported heterogeneous Cu(i) catalysis for CuAAC reactions but only for the preparation of non-conjugated systems. It is well known^6a,7a,b^ that carrying out a reaction using a heterogeneous catalyst, product purification can be simplified because of the facile recovery from the reaction mixture by filtration/centrifugation. In addition, in most cases the need of chromatography for metal removal may be avoided, thus reducing the operation cost and the waste associated with the process. Heterogeneous catalysts are also easy to handle and are usually recyclable and safer to be stored/discarded. Another fundamental question is the control over selectivity in the click polymerization when using a broader range of monomers to access different conjugated polymers. Further efforts in this direction are certainly needed to achieve a more mature and broader scope synthetic methodology. Among the possible routes for optimization, the proper design of monomers, catalysts, and/or process conditions appear to be promising strategies. Expanding the range of azide/alkyne monomer substituents to broaden the development of CuAAC click-polymerization towards conjugated polymers is also desirable.
